# Radiological diagnosis of brain radiation necrosis after cranial irradiation for brain tumor: a systematic review

**DOI:** 10.1186/s13014-019-1228-x

**Published:** 2019-02-06

**Authors:** Motomasa Furuse, Naosuke Nonoguchi, Kei Yamada, Tohru Shiga, Jean-Damien Combes, Naokado Ikeda, Shinji Kawabata, Toshihiko Kuroiwa, Shin-Ichi Miyatake

**Affiliations:** 1 Department of Neurosurgery, Osaka Medical College, 2-7, Daigakumachi, Takatsuki, Osaka, 569-8686 Japan; 2 Department of Radiology, Kyoto Prefectural University of Medicine, Kyoto, Japan; 3 Department of Nuclear Medicine, Hokkaido University Graduate School of Medicine, Sapporo, Japan; 4 Infections and Cancer Epidemiology Group, International Agency for Research on Cancer, World Health Organization, Lyon, France

**Keywords:** Brain tumor, Diagnosis, Radiation necrosis, Radiology, Recurrence

## Abstract

**Introduction:**

This systematic review aims to elucidate the diagnostic accuracy of radiological examinations to distinguish between brain radiation necrosis (BRN) and tumor progression (TP).

**Methods:**

We divided diagnostic approaches into two categories as follows—conventional radiological imaging [computed tomography (CT) and magnetic resonance imaging (MRI): review question (RQ) 1] and nuclear medicine studies [single photon emission CT (SPECT) and positron emission tomography (PET): RQ2]—and queried. Our librarians conducted a comprehensive systematic search on PubMed, the Cochrane Library, and the Japan Medical Abstracts Society up to March 2015. We estimated summary statistics using the bivariate random effects model and performed subanalysis by dividing into tumor types—gliomas and metastatic brain tumors.

**Results:**

Of 188 and 239 records extracted from the database, we included 20 and 26 studies in the analysis for RQ1 and RQ2, respectively. In RQ1, we used gadolinium (Gd)-enhanced MRI, diffusion-weighted image, MR spectroscopy, and perfusion CT/MRI to diagnose BRN in RQ1. In RQ2, ^201^Tl-, ^99m^Tc-MIBI-, and ^99m^Tc-GHA-SPECT, and ^18^F-FDG-, ^11^C-MET-, ^18^F-FET-, and ^18^F-BPA-PET were used. In meta-analysis, Gd-enhanced MRI exhibited the lowest sensitivity [63%; 95% confidence interval (CI): 28–89%] and diagnostic odds ratio (DOR), and combined multiple imaging studies displayed the highest sensitivity (96%; 95% CI: 83–99%) and DOR among all imaging studies. In subanalysis for gliomas, Gd-enhanced MRI and ^18^F-FDG-PET revealed low DOR. Conversely, we observed no difference in DOR among radiological imaging in metastatic brain tumors. However, diagnostic parameters and study subjects often differed among the same imaging studies. All studies enrolled a small number of patients, and only 10 were prospective studies without randomization.

**Conclusions:**

Differentiating BRN from TP using Gd-enhanced MRI and ^18^F-FDG-PET is challenging for patients with glioma. Conversely, BRN could be diagnosed by any radiological imaging in metastatic brain tumors. This review suggests that combined multiparametric imaging, including lesional metabolism and blood flow, could enhance diagnostic accuracy, compared with a single imaging study. Nevertheless, a substantial risk of bias and indirectness of reviewed studies hindered drawing firm conclusion about the best imaging technique for diagnosing BRN.

**Electronic supplementary material:**

The online version of this article (10.1186/s13014-019-1228-x) contains supplementary material, which is available to authorized users.

## Introduction

The pathology of progressive brain radiation necrosis (BRN) primarily includes inflammation and angiogenesis in which cytokines, chemokines, and vascular endothelial growth factor are upregulated [1–7]. Inflammation and angiogenesis account for the breakdown of the blood–brain barrier, resulting in contrast-enhanced lesions and perilesional edema. Nevertheless, recurrent tumors also displayed these findings on computed tomography (CT) and magnetic resonance image (MRI). Distinguish between BRN and tumor progression (TP) is rather challenging on conventional radiological imaging. In addition, surgical removal of tissue samples is invasive even in cases of stereotactic biopsies, although pathological diagnosis remains the gold standard. Moreover, needle biopsy poses a risk of misdiagnosis because BRN is typically a heterogeneous lesion, with coexisting radiation necrosis and tumor cells [8]. Ideally, BRN is diagnosed by relatively less-invasive radiological examinations that evaluate the whole lesion, compared with needle biopsy. Recently, bevacizumab was shown to markedly reduce brain edema and improve patients’ clinical statuses, and is a promising and novel treatment for BRN [9–12]. As bevacizumab delays the surgical wound healing, patients diagnosed with BRN by surgical biopsy need to wait for wound healing before the bevacizumab administration. However, bevacizumab could be administered immediately after the diagnosis of BRN by noninvasive radiological imaging studies.

The last several decades have witnessed an upsurge of various functional images and nuclear medicine studies that have developed and seem useful for differentiating between BRN and TP. For example, MR spectroscopy (MRS) and diffusion-weighted images (DWI) offer qualitative data without using contrast media. Perfusion images depict cerebral blood flow or volume (CBV) using contrast media. In addition, single photon emission CT (SPECT) and positron emission tomography (PET) display metabolic data using various tracers. Despite these radiological imaging studies being useful for differentiating between BRN and TP, it remains unclear which imaging study is preferable. Hence, this systematic review aims to illustrate the diagnostic accuracy of radiological imaging for differentiation between BRN and TP.

## Methods

### Search strategy

We conducted a systematic review based on the directives of the Preferred Reporting Items for Systematic Reviews and Meta-Analysis statement (PRISMA) [13]. Our review question (RQ) was structured using the patient, exposure, comparison, and outcome (PECO) approach. Our RQ was, “Are radiological imaging studies useful for distinguishing BRN from TP in brain tumor patients treated with radiotherapy who exhibit clinical or radiological disease progression?” Regarding radiological examinations, although many hospitals own CT and MRI equipment, SPECT and PET are less common. Hence, we categorized the radiological examinations into the following two groups: CT and MRI as *conventional radiological imaging* (RQ1) and SPECT and PET as *nuclear medicine imaging* (RQ2). Our medical librarians conducted a comprehensive systematic search using the PubMed, Cochrane Library, and Japan Medical Abstracts Society databases, up to March 2015. Additional file 1 presents the keywords used to complete the search. Regarding PET, several new tracers have been developed in recent years; however, these are too early to assess the diagnostic ability of differentiation between BRN and TP because numerous studies are required for systematic review. Hence, “fluorodeoxyglucose”/“FDG” and “amino acid”/“methionine” were included in the keywords. These tracers have been used since long, and an adequate number of studies are expected to be identified for the systematic review. Two reviewers (MF and KY for RQ1, and NN and TS for RQ2) screened and determined studies to be included for each RQ. Eligible studies investigated the diagnostic accuracy of radiological imaging methods for differentiation between BRN and TP and were written in English or Japanese. Eligible participants were patients who underwent radiotherapy for brain tumors. However, we excluded case reports, letters to the editor, and conference abstracts, as well as studies without sufficient information for construction of a 2 × 2 table.

### Quality assessment and data analysis

The reviewers assessed the quality of individual studies using the Quality Assessment of Diagnostic Accuracy Studies 2 (QUADAS-2) checklist [14]. The QUADAS-2 tool comprises four domains as follows: patient selection; index test; reference standard; and flow and timing. QUADAS-2 segregates study quality into “risk of bias” and “applicability.” We judged the risk of bias using signaling questions and applicability by concerns that the study does not match the RQ. Each domain was assessed in terms of the risk of bias and, the first three domains were also assessed in terms of concerns about applicability. Furthermore, the risk of bias and applicability were assessed by reviewers in each RQ. Besides QUADAS-2 assessment, indirectness, inconsistency, and imprecision were also assessed for the body of evidence.

We used Cochrane Collaboration Review Manager 5 (Review Manager. Version 5.3. Copenhagen: The Nordic Cochrane Centre, The Cochrane Collaboration, 2014) to analyze the data of each study. The sensitivity, specificity, and accuracy, as well as 95% confidence intervals (CI), were calculated and evaluated using visual inspection of forest plots. In the quantitative synthesis, we completed bivariate diagnostic random effect meta-analysis and summary receiver operating characteristic (SROC) curves with R Software version 3.4.3 (https://www.R-project.org/) using mada package including “reitsma” function (https://www.rdocumentation.org/packages/mada/versions/0.5.8/topics/reitsma) to produce summary estimates for the sensitivity and specificity [15] and “madauni” (https://www.rdocumentation.org/packages/mada/versions/0.5.8/topics/madauni) for diagnosis odds ratio (DOR), provided by CRAN (The Comprehensive R Archive Network; https://cran.r-project.org/). Furthermore, a subanalysis of the quantitative synthesis was performed, dividing into tumor types, gliomas and metastatic brain tumors.

## Results

### Search results

**Fig. 1:**
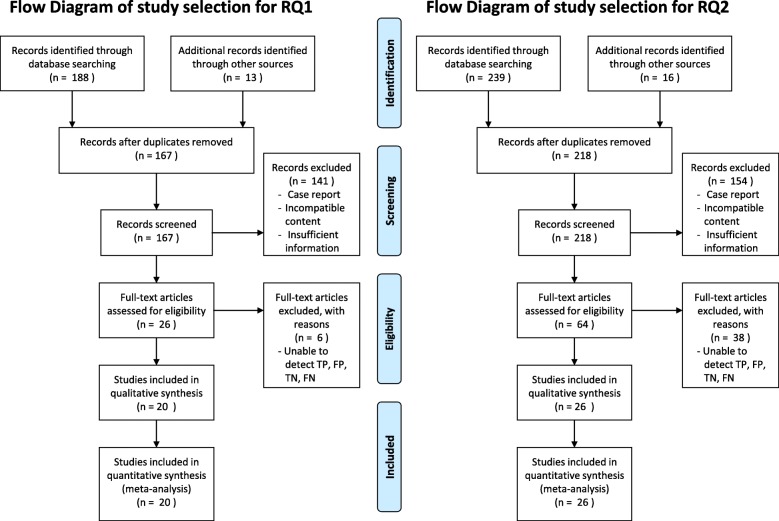
Flow diagrams of the study selection for RQ1 (conventional radiological imaging) and RQ2 (nuclear medicine imaging)

Our database search for RQ1 yielded 188 papers. In addition, 13 records were identified from literature reviews. Of 201 papers, we excluded 34 because of duplication and 141 because they were case reports, featured incompatible contents, or had inadequate information. In the first screening, we identified 26 papers for full-text assessment. In the second screening, six papers were excluded because we could not identify the numbers of patients with true/false positive and negative results, or papers where a 2 × 2 table could not be constructed. Finally, we included 20 studies in the qualitative synthesis (Fig. 1; Table 1). The database search for RQ2 yielded 239 papers. In addition, 16 papers were identified from review articles. Of 255 papers, we excluded 37 because of duplication and 154 because of case reports, incompatible contents, or inadequate information. We selected 64 papers for the full-text screening; of these, 38 papers were excluded because of the inability of a 2 × 2 table construction. Finally, we selected 26 studies in the RQ2 meta-analysis (Fig. 1; Table 2).

**Table 1:** Summary of studies for CQ1 (conventional radiological imaging)

References	Study Design	Patient	Exposure	Comparison	Outcome	Reference Standard
Dequesada 2008 [16]	Retrospective case series	32 Mets treated with SRS	MRI *lesion quotient ≤ 0.3* (retrospective) (blinded review)	AV shunt, enhancement pattern, etc.	Sensitivity: 80% Specificity: 96.4% Accuracy: 94%	Histology for all 32 lesions (blinded review)
Leeman 2013 [17]	Retrospective case series	49 Mets 52 lesions treated with SRS	MRI *edema/lesion volume ratio ≥ 10* (retrospective) (blinded review)	None	Sensitivity: 84.6% Specificity: 62.9% Accuracy: 69%	Histology obtained by removal in all 52 lesions
Santra 2011 [18]	Prospective cohort study	85 gliomas (16 GIVs, 28 GIIIs, 37GIIs, 4 GIs)	MRI *Gd-enhancement* (blinded review)	^99m^Tc-GHA-PET (blinded review)	Sensitivity: 24.1% Specificity: 94.6% Accuracy: 71%	5 Histological and 80 clinical diagnosis (repeat imaging, F/U ≥ 6 mos)
Tie 2008 [19]	Retrospective case series (consecutive)	19 gliomas (21 examinations) (7 GBMs, 7 AAs, 5 AOs)	MRI *T1, T2, FLAIR, GdT1 Radiological report*	^201^Tl-SPECT	Sensitivity: 75% Specificity: 64.7% Accuracy: 67%	9 Histological and 12 clinical diagnosis (clinical course)
Di Costanzo 2014 [20]	Retrospective case series (consecutive)	29 GBMs	DWI *ADC alone (higher)*		Sensitivity: 87.5% Specificity: 81.0% Accuracy: 83%	Clinical diagnosis (≥4 F/U MRI, 2–6-mo interval) in all 29 cases
			MRS *Cho/Crn alone (lower)*		Sensitivity: 75.0% Specificity: 81.0% Accuracy: 79%	
			PWI *rCBV alone (lower)*		Sensitivity: 87.5% Specificity: 85.7% Accuracy: 86%	
			MRS, DWI, and MRP *Cho/Chon, ADC, CBV*		Sensitivity: 100% Specificity: 95.2% Accuracy: 97%	
Cha 2013 [21]	Retrospective case series (consecutive)	16 Mets treated with SRS	DWI *3 layer pattern of ADC*		Sensitivity: 100% Specificity: 87.5% Accuracy: 94%	Histological diagnosis by removal in all 16 cases
			MRP *rCBV ≤ 4.1* (retrospective)		Sensitivity: 100% Specificity: 71.4% Accuracy: 88%	
			DWI and perfusion MRI *3 layer pattern ADC* *with rCBV ≤ 2.6 or rCBV ≤ 4.1* (retrospective)		Sensitivity: 100% Specificity: 100% Accuracy: 100%	
Amin 2012 [22]	Retrospective case series	24 primary brain tumors (7 GBMs, 12 AAs, 5 GI-IIAs)	MRS *Cho/Cr < 1.5 and Cho/NAA < 1* (prospective)	^99m^Tc-DMSA-PET	Sensitivity: 100% Specificity: 61.1% Accuracy: 71%	5 Histological (5B) and 19 clinical diagnosis (clinical course and F/U image)
Ando 2004 [23]	Retrospective case series	20 gliomas (10 GBMs, 2 AAs, 1 OD, 7 GI-IIAs)	MRS *Cho/Cr < 1.5* (retrospective)	None	Sensitivity: 83.3% Specificity: 64.3% Accuracy: 70%	7 Histological and 13 clinical diagnosis (MRI F/U ≥ 1 year)
Elias 2011 [24]	Retrospective case series	27 intracranial neoplasms	MRS *Cho/NAA < 0.92* (Retrospective)	MRS *Higher NAA/Cr, lower Cho/nNAA*	Sensitivity: 90% Specificity: 86.7% Accuracy: 88%	10 Histology and15 clinical diagnosis (3–6-mo F/U imaging)
Huang 2011 [25]	Retrospective case series	33 metastatic lesions	MRS *24 multivoxel MRS Cho/nCho ≤ 1.2* (retrospective)		Sensitivity: 100% Specificity: 33.3% Accuracy: 48%	4 Histological and 29 clinical diagnosis (F/U image)
			MRP *rCBV ≤ 2* (retrospective)		Sensitivity: 100% Specificity: 55.6% Accuracy: 70%	
Nakajima T 2009 [26]	Retrospective case series	18 gliomas (8 GBMs, 6AAs, 4 DAs)	MRS *Lac/Cho > 1.05* (retrospective)	MET-PET	Sensitivity: 88.9% Specificity: 100% Accuracy: 94%	14 Histological and 4 clinical diagnosis (clinical course and F/U image ≥6 mos)
Peca 2009 [27]	Retrospective case series	15 GBMs after Stupp protocol	MRS *neither increased Cho nor decreased NAA*	None	Sensitivity: 25% Specificity: 100% Accuracy: 80%	10 Histological and 5 clinical diagnosis (clinical and 3-mo interval MRI F/U)
Zeng IJROBP 2007 [28]	Prospective cohort study	55 HGGs (36 GIIIs, 19 GIVs)	MRS *Lower Cho/Cr and lower Cho/NAA*		Sensitivity: 100% Specificity: 93.8% Accuracy: 96%	39 Histological (10B, 29R) and 16 clinical diagnosis (MRI F/U ≤ 22 mos)
			Proton MRS and DWI *combination of Cho/Cr, Cho/NAA, ADC ratio (higher)*		Sensitivity: 100% Specificity: 93.8% Accuracy: 96%	
Zeng JNO 2007 [29]	Prospective cohort study	28 HGGs (20 GIIIs, 8 GIVs)	proton MRS *Cho/Cr < 1.71, Cho/NAA < 1.71* (retrospective)	None	Sensitivity: 100% Specificity: 94.1% Accuracy: 96%	21 Histological (5B, 16R) and 7 clinical diagnosis (F/U MRI)
Jain 2011 [30]	Retrospective case series	38 brain tumors	PCT *rCBV ≤ 1.5* *PS ≤ 2.5* (retrospective)	None	Sensitivity: 90.9, 81.8% Specificity: 81.5, 81.5% Accuracy: 84, 82%	Histological diagnosis in all 38 cases
Barajas 2009 [31]	Retrospective case series	57 GBMs (66 examinations)	MRP *rPH < 1.38* (retrospective) (blinded review)	None	Sensitivity: 80% Specificity: 89.1% Accuracy: 86%	64 Histological (62R, 2B) and 2 clinical diagnosis (MRI F/U ≥ 22 mos)
Bisdas 2011 [32]	Prospective cohort study	18 HGGs	MRP *K*^*trans*^ *≤ 0.19* (retrospective)	None	Sensitivity: 83.3% Specificity: 100% Accuracy: 94%	5 Histological and 13 clinical diagnosis (MRI F/U ≥ 6 mos)
Bobek-Billewicz 2011 [33]	Retrospective case series	8 gliomas (11 lesions) (2 GBMs, 5 AAs, 1 DA)	MRP *Normalized CBVmean ≤ 1.25* (retrospective)	DWI	Sensitivity: 100% Specificity: 60% Accuracy: 82%	8 Histological and 3 clinical diagnosis (F/U image)
Kim 2010 [34]	Retrospective case series	10 HGGs (5 GBMs, 3 AAs, 2 AOs)	MRP *normalized rCBV ≤ 3.69* (retrospective)	^18^F-FDG-PET, ^11^C-MET-PET	Sensitivity: 100% Specificity: 100% Accuracy: 100	3 Histological (3 R) and 7 clinical diagnosis (3-mo interval MRI F/U of 28 mos)
Narang J 2011 [35]	Retrospective case series	29 brain tumors (24 PBTs, 5 Mets)	MRP *nMSIVP < 0.031* *(MSIVP ≤ 9.5)* (retrospective)	None	Sensitivity: 77.8% Specificity: 95% Accuracy: 90%	20 Histological and 9 clinical diagnosis (imaging and clinical F/U ≦13 mos)

**Table 2:** Summary of studies for CQ2 (nuclear medicine imaging)

References	Study Design	Patient	Exposure	Comparison	Outcome	Reference standard
Tie 2008 [19]	Retrospective case series (consecutive)	19 HGGs (7 GBMs, 7 AAs, 5 AOs) (21 exams)	^201^Tl-SPECT *Visual assessment*	MRI	Sensitivity 100.0% Specificity 82.4% Accuracy 85.7%	9 Histological and12 clinical diagnosis (clinical and MRI F/U ≦6 mos)
Gomez-Rio 2008 [36]	Prospective cohort study	Gliomas (44 HGGs, 32 LGGs)	^201^Tl-SPECT *Visual assessment* (blind review)	Tl-SPECT + MRI vs FDG-PET + MRI	Sensitivity 85.7% Specificity 92.7% Accuracy 90.8%	23 Histological and 53 clinical diagnosis (F/U image)
Kahn 1994 [37]	Prospective cohort study	17 Gliomas, 1 Met, 1 esthesioblastoma	^201^Tl-SPECT *Tl index*		Sensitivity 40.0% Specificity 68.8% Accuracy 61.9%	5 Histological and 14 clinical diagnosis (clinical F/U) (blinded review)
			^18^F-FDG-PET *PET grade (visual assessment)*		Sensitivity 40.0% Specificity 81.3% Accuracy 71.4%	
Matsunaga 2013 [38]	Retrospective case series	27 Gliomas, 48 Mets (107 lesions)	^201^Tl-SPECT *Retention index ≤0.775* (retrospective)	None	Sensitivity 83.3% Specificity 83.1% Accuracy 83.2%	19 Histological and 88 clinical diagnosis (clinical and MRI F/U)
Stokkel 1999 [39]	Prospective cohort study	16 Gliomas	^201^Tl-SPECT *Tl uptake index*		Sensitivity 100.0% Specificity 100.0% Accuracy 100.0%	2 Histological and14 clinical diagnosis (clinical and imaging F/U of 12 mos)
			^18^F-FDG-PET *FDG grade (5-point scale)*		Sensitivity 100.0% Specificity 66.7% Accuracy 75.0%	
Yamamoto 2002 [40]	Retrospective case series	14 Gliomas, 4 Mets, 1 ML, 1 MM, 1 HPC	^201^Tl-SPECT *L/N < 2.4* (retrospective)		Sensitivity 83.3% Specificity 93.3% Accuracy 90.5%	10 Histological and 11 clinical diagnosis (F/U MRI for 10 mos)
			Tc-MIBI –SPECT *L/N < 5.89* (retrospective)		Sensitivity 83.3% Specificity 93.3% Accuracy 90.5%	
Le Jeune 2006 [41]	Retrospective case series	81 Gliomas	Tc-MIBI –SPECT *L/N < 2.0* (retrospective)	None	Sensitivity 93.2% Specificity 90.3% Accuracy 91.5%	14 Histological (14 B) and 67 clinical diagnosis (clinical and image F/U ≥ 6 mos)
Barai 2004 [42]	Retrospective case series (consecutive)	73 Glioma	^99m^Tc-GHA-SPECT *GHA index (L/N)* *< 2.0* (retrospective) (blind review)	None	Sensitivity 81.0% Specificity 98.1% Accuracy 93.2%	Clinical diagnosis (clinical F/U) in all 73 patients
Belohlávek 2003 [43]	Retrospective case series (consecutive)	25 Mets (57 lesions)	^18^F-FDG-PET *Visual assessment* (blind review)	MRI	Sensitivity 93.9% Specificity 75.0% Accuracy 92.2%	3 Histological and 54 clinical diagnosis (clinical and imaging F/U ≤ 26 weeks)
Chao 2001 [44]	Retrospective case series	15 Glioma, 32 Mets 44 lesions (8 glioma, 36 Mets)	^18^F-FDG-PET *Visual assessment*	None	Sensitivity 81.3% Specificity 75.0% Accuracy 77.3%	17 Histological and 27 clinical diagnosis (imaging F/U of 5.6 mos)
Horky 2011 [45]	Retrospective case series (consecutive)	32 Mets 25 patients with 27 lesions, 28 scans	^18^F-FDG-PET *L/N SUVmax change over time (ROC cutoff ≤ 0.19)* (retrospective)	None	Sensitivity 100.0% Specificity 94.7% Accuracy 96.7%	17 Histological and 13 clinical diagnosis (MRI F/U ≥ 6 mos)
Karunanithi 2013 [46]	Prospective cohort study	28 Gliomas	^18^F-FDG-PET *Visual assessment (T/W ratio ≤ 0.9)* (retrospective) (blind review)	^18^F-DOPA-PET	Sensitivity 100.0% Specificity 47.6% Accuracy 60.7%	4 Histological and 24 clinical diagnosis (clinical and imaging F/U)
Ozsunar 2010 [47]	Prospective cohort study	30 Gliomas 26 PET evaluations	^18^F-FDG-PET *Visual assessment* (blind review)	ASL imaging, DSCE-CBV imaging	Sensitivity 90.0% Specificity 81.3% Accuracy 84.6%	Histological diagnosis in all 35 evaluations
Takenaka 2014 [48]	Retrospective case series (consecutive)	50 Gliomas	^18^F-FDG-PET *L/N ratio ≤ 1.26* (retrospective)	^11^C-Cho-PET	Sensitivity 75.0% Specificity 76.5% Accuracy 76.0%	Histological diagnosis in all 50 patients
			^11^C-MET-PET *L/N ratio ≤ 2.51* (retrospective)	^11^C-Cho-PET	Sensitivity 87.5% Specificity 91.2% Accuracy 90.0%	
Tan 2011 [49]	Retrospective case series	37 Gliomas, 15 Mets, 1 neuroblastoma, 1 lymphoma, 1 germinoma	^18^F-FDG-PET *visual assessment*	^11^C-Cho-PET	Sensitivity 62.5% Specificity 76.9% Accuracy 72.7%	17 Histological and 38 clinical diagnosis (3-m interval MRI F/U ≥ 11 mos)
Okamoto 2011 [50]	Retrospective case series	29 Gliomas and Mets 33 lesions	^11^C-MET-PET *L/N ratio ≤ 1.4* (retrospective)	None	Sensitivity 90.0% Specificity 91.3% Accuracy 90.9%	14 Histological and 19 clinical diagnosis (MRI over 2 yrs)
Tsuyuguchi 2004 [51]	Retrospective case series	11 HGGs (8 GBMs, 3 AAs)	^11^C-MET-PET *Visual assessment*	Health volunteers	Sensitivity 100.0% Specificity 60.0% Accuracy 81.8%	8 Histological and 3 clinical diagnosis (clinical and MRI F/U ≥ 5 mos)
Yamane 2010 [52]	Retrospective case series (consecutive)	80 brain neoplasms (47scans)	^11^C-MET-PET *visual assessment*	None	Sensitivity 100.0% Specificity 88.1% Accuracy 89.4%	30 Histological and 34 clinical diagnosis (clinical and imaging F/U of 435 days)
Terakawa 2008 [53]	Retrospective case series	26 Gliomas, 51 Mets 88 PETs	^11^C-MET-PET *L/Nmean ratio* *Met ≤ 1.41* *Glioma ≤ 1.58* (retrospective)	None	Sensitivity 75.0% Specificity 77.5% Accuracy 76.1%	44 Histological and 44 clinical diagnosis (MRI F/U ≥ 6mos)
Saginoya 2012 [54]	Retrospective case series	14 gliomas, 23 Mets, 2 lymphoma (49 scans)	^11^C-MET-PET *L/N ratio ≤ 1.33* (retrospective)	None	Sensitivity 100.0% Specificity 72.0% Accuracy 85.7%	Histological and clinical diagnosis (imaging F/U ≥ 6 mos)
Kawai 2008 [55]	Retrospective case series	11 HGGs (13 scans), 14 Mets (15 scans)	^11^C-MET-PET *SUVmax ≤ 2.5* (glioma) (retrospective)	^18^F-FLT-PET	Sensitivity 77.8% Specificity 76.9% Accuracy 77.3%	12 histological and 10 clinical diagnosis (MRI F/U ≥ 1 yr)
Sunada 2001 [56]	Retrospective case series	26 Mets (33 lesions)	^11^C-MET-PET *visual assessment, T/N ratio*	None	Sensitivity 83.3% Specificity 100.0% Accuracy 90.9%	7 histological and 26 clinical diagnosis (imaging F/U ≥ 6 mos)
Pӧpperl 2004 [57]	Retrospective case series	53 Gliomas (27 GIVs, 16 GIIIs, 9 GIIs, 1 GI)	^18^F-FET-PET *SUVmax/BG ratio ≤ 2.0* (retrospective)	None	Sensitivity 100.0% Specificity 100.0% Accuracy 100.0%	27 histological and 26 clinical diagnosis (clinical F/U of 34 mos)
Rachinger 2005 [58]	Retrospective case series (consecutive)	45 Gliomas (22 GIVs, 12 GIIIs, 10 GIIs, 1 GI)	^18^F-FET-PET *SUV MAX ≤ 2.2* (prospective)	MRI	Sensitivity 92.9% Specificity 100.0% Accuracy 97.8%	32 histological and 13 clinical diagnosis (clinical F/U)
Galldiks 2012 [59]	Retrospective case series (consecutive)	31 Mets (40 lesions)	^18^F-FET-PET *TBR(tumor-to-brain ratio) mean ≤ 1.95* (retrospective)	None	Sensitivity 90.5% Specificity 73.7% Accuracy 82.5%	11 histological and 29 clinical diagnosis (clinical and MRI F/U of 12 mos)
Miyashita 2008 [60]	Retrospective case series	38 Gliomas, 2 Mets, 2 Head and Neck cancers (49 scans)	^18^F-BPA-PET *L/Nmean ratio ≤ 2.5* (retrospective)	None	Sensitivity 100.0% Specificity 97.2% Accuracy 98.0%	44 histological and 5 clinical diagnosis (MRI F/U > 4 mos)

### Meta-analysis

**Fig. 2:**
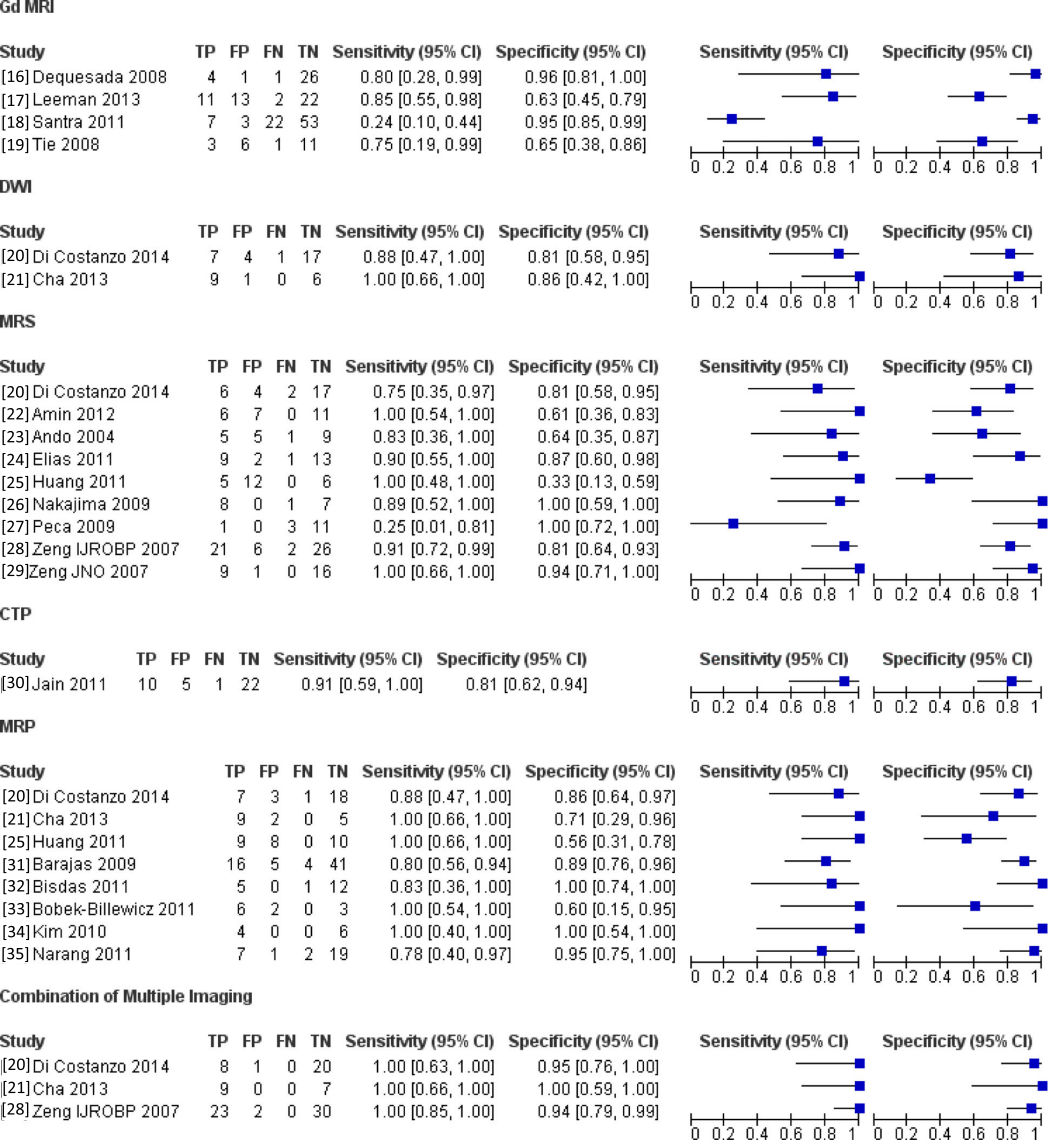
The forest plot of each study for RQ1 (conventional radiological imaging)

**Fig. 3:**
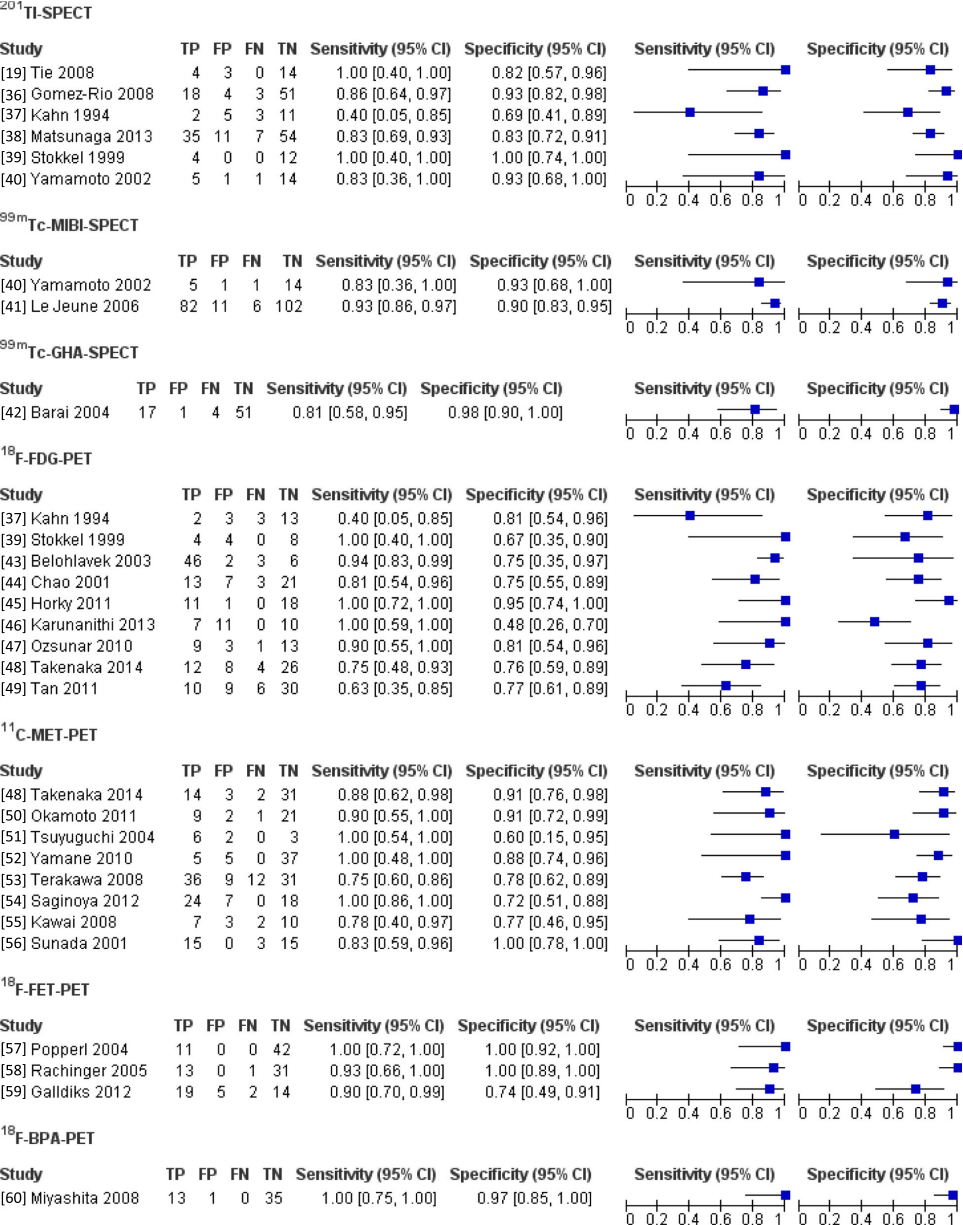
The forest plot of each study in RQ2 (nuclear medicine imaging)

For RQ1, gadolinium (Gd)-enhanced MRI, DWI, MRS, and CT/MR perfusion were identified as methods to diagnose BRN. The Gd-MRI analysis was included four studies [16–19], the DWI analysis was included in two studies [20, 21], and the MRS analysis was included nine studies [20, 22–29]. The CT and MRI perfusion analyses were included in 1 [30] and eight studies [20, 21, 25, 31–35]. In these studies, the combination of multiple imaging (DWI and MRS, DWI and perfusion MRI, or DWI, MRS, and perfusion MRI) was also evaluated in three studies [20, 21, 28]. Additional file 2 describes the characteristics of studies included in the analysis of each modality. Figure 2 shows forest plots of each study in RQ1. In 26 studies for RQ2, SPECT, with a tracer of ^201^Tl, ^99m^Tc-methoxyisobutylisonitrile (MIBI), and ^99m^Tc-glucoheptonate (GHA), and PET, with a tracer of ^18^F-fluorodeoxyglucose (FDG), ^11^C-methionine (MET), ^18^F-fluoroethyltyrosine (FET), and ^18^F-boronophenylalanine (BPA), were used to differentiate between BRN and TP. The analyses of ^201^Tl-, ^99m^Tc- MIBI-, and ^99m^Tc- GHA-SPECT included six studies [19, 36–40], two studies [40, 41], and one study [42], respectively. The analyses of ^18^F-FDG-, ^11^C-MET-, ^18^F-FET-, and ^18^F-BPA-PET included nine studies [37, 39, 43–49], eight studies [48, 50–56], three studies [57–59], and one study [60], respectively. Additional file 2 describes information about each study. Figure 3 shows forest plots of RQ2 study.

**Fig. 4:**
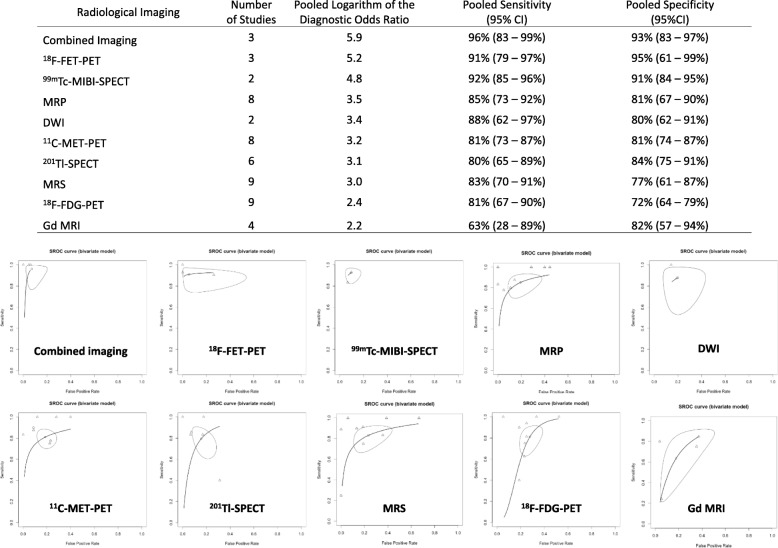
Pooled estimates of the diagnostic accuracy and summary receiver operating characteristic curves of the radiological imaging in all included studies

Figure 4 shows the pooled estimates of the diagnostic accuracy and SROC curves of the radiological imaging techniques. Combined imaging (DWI and MRS, DWI and perfusion MRI, or DWI, MRS, and perfusion MRI) exhibited the highest sensitivity (96%; 95% CI: 83–99%), and ^18^F-FET-PET exhibited the highest specificity (95%; 95% CI: 61–99%), resulting in high DORs. Conversely, the sensitivity of Gd-enhanced MRI was the lowest (63%; 95% CI: 28–89%), and the specificity of ^18^F-FDG-PET was the lowest (72%; 95% CI: 64–79%), which contributed to low DORs. Although the DOR of combined imaging (DWI and MRS, DWI and perfusion MRI, or DWI, MRS, and perfusion MRI) was the highest among all radiological imaging techniques, the DORs of perfusion MRI, DWI, and MRS were not high (MRP: 3.5, DWI: 3.4, and MRS: 3.0; Fig. 4).

**Fig. 5:**
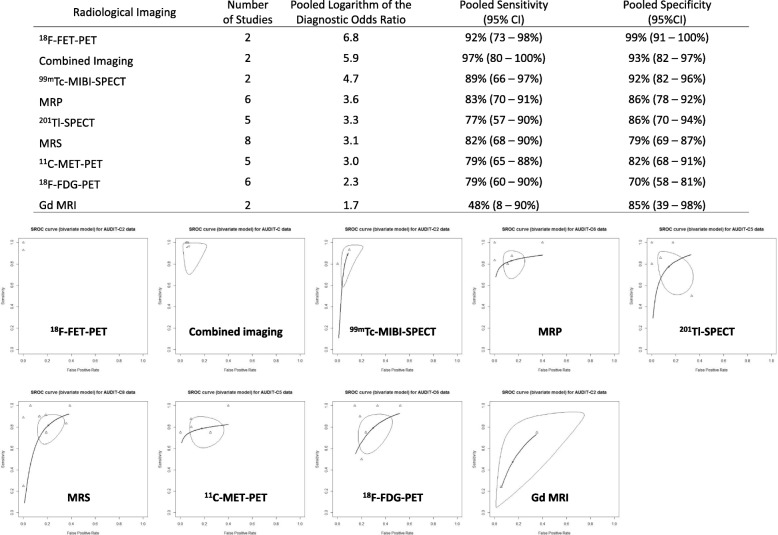
Pooled estimates of the diagnostic accuracy and summary receiver operating characteristic curves of the radiological imaging in studies for gliomas

**Fig. 6:**
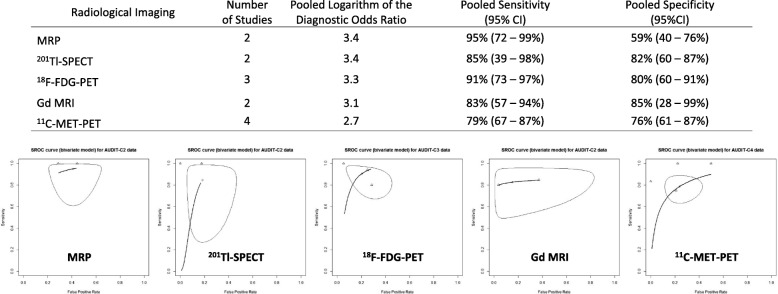
Pooled estimates of the diagnostic accuracy and summary receiver operating characteristic curves of the radiological imaging in studies for metastatic brain tumors

In the subanalysis dividing into tumor types, gliomas and metastatic brain tumors, 23 studies included only gliomas and eight studies included only metastatic brain tumors. In addition, 14 studies included patients with various brain tumors; of these, 9 studies could be categorized into patients with glioma and patients with metastatic brain tumors. Excluding radiological imaging with a single study, Gd-enhanced MRI, MRS, perfusion, MRI, combined imaging (DWI and MRS, DWI and perfusion MRI, or DWI, MRS, and perfusion MRI), SPECT with ^201^Tl and ^99m^Tc, and PET with ^18^F-FDG, ^11^C-MET, and ^18^F-FET were quantitatively synthesized in the subanalysis for gliomas (Fig. 5). Combined imaging (DWI and MRS, DWI and perfusion MRI, or DWI, MRS, and perfusion MRI) exhibited the highest sensitivity (97%; 95% CI: 80–100%), and ^18^F-FET-PET exhibited the highest specificity (99%; 95% CI: 91–100%), which resulted in higher DORs among radiological imaging for gliomas. Conversely, Gd-enhanced MRI and ^18^F-FDG-PET exhibited the lowest sensitivity (48%; 95% CI: 8–90%) and specificity (70%; 95% CI: 58–81%), respectively, among imaging for gliomas; these 2 studies had low DORs. In the subanalysis of metastatic brain tumors, Gd-enhanced MRI, perfusion MRI, ^201^Tl-SPECT, ^18^F-FDG-, and ^11^C-MET-PET were included in the meta-analysis (Fig. 6). Perfusion MRI exhibited the highest sensitivity (95%; 95% CI: 72–99%) but the lowest specificity (59%; 95% CI: 40–76%) among imaging for metastatic brain tumors. Thus, DORs were almost the same among these 5 imaging methods. Comparing between gliomas and metastatic brain tumors, Gd-enhanced MRI and ^18^F-FDG-PET declined the diagnostic accuracy of differentiating between BRN and TP in patients with glioma than that in patients with metastatic brain tumors. However, we observed no difference in the diagnostic accuracy between gliomas and metastatic brain tumors in perfusion MRI, ^201^Tl-SPECT, and ^11^C-MET-PET.

### Quality assessment

**Fig. 7:**
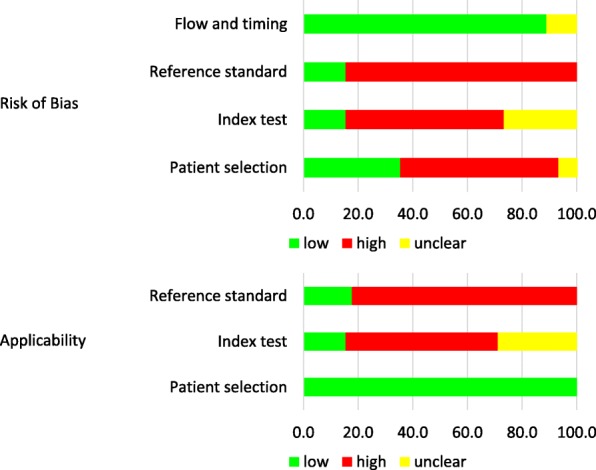
Clustered bar graphs of quality results on the QUADAS-2 criteria tool

In this study, we assessed the risk of bias in accordance with QUADAS-2 (Fig. 7). Regarding patient selection, no randomized studies were included in our research results. While nine prospective cohort studies were identified [18, 28, 29, 32, 36, 37, 39, 46, 47], the remaining 36 studies were retrospective. Of 36 retrospective studies, patients were consecutively enrolled in 10 studies [19–21, 42, 43, 45, 48, 52, 58, 59]. In the index testing, the cutoff values of diagnostic parameters were preset and prospectively assessed in two studies but without blinding [22, 58]. In addition, cutoff values of diagnostic parameters were retrospectively exhibited with the diagnostic accuracy in other 28 studies; of these 28 studies, the cutoff values of diagnostic parameters were blindly measured in only five studies [16, 17, 31, 42, 46]. Only six studies used histopathology as the reference standard for all patients [16, 17, 21, 30, 47, 48], while two studies adopted clinical diagnosis as the reference standard [20, 42]. The remaining studies used the clinical diagnosis as the reference standard for some patients; in these studies, the clinical diagnosis was obtained from clinical and imaging follow-up. Of note, radiation necrosis was diagnosed if the clinical course was stable, and/or if the tumor was stable or shrunk or disappeared on a follow-up image. In most studies, the follow-up period was > 6 months. Only one study blindly reviewed the reference standard [16]. Regarding the applicability, patient selection was applicable to the RQ, but a nonblinded review of index tests and retrospectively-set cutoff values were not applicable to the RQ because of a high risk of bias-favoring index tests. Furthermore, studies that included clinical diagnosis as the reference standard had a high risk of bias and were not applicable to the RQ because radiological imaging data were usually included for clinical diagnosis.

Several factors were associated with indirectness. As mentioned in the subanalysis, various brain tumors were included in the studies. Regarding the index test, parameters and cutoff values were different among studies with the same imaging modality. Notably, six different parameters were used among studies for MRS, and four different parameters were used among studies for perfusion MRI. Regarding cutoff values, the L/N ratio was mostly used in four studies with ^11^C-MET-PET; however, cutoff values were different among these studies. Studies with Gd-MRI, MRS, ^201^Tl-SPECT, and ^18^F-FDG-PET reported inconsistency in the sensitivity. In these imaging studies, one study revealed low sensitivity unlike the remaining studies reporting high sensitivity. In this review, most of the included studies had a large 95% CI as imprecision because of the small sample size. Notably, 33 (71.7%) studies included patients/lesions/scans < 50, and only one study included lesions > 100. The small sample size could be a bias to include specific patients only.

## Discussion

The meta-analysis revealed a trend that the sensitivity was generally higher than the specificity in all radiological imaging methods; that is, TP was occasionally misdiagnosed as BRN by these imaging methods. ^18^F-FET-PET and ^99m^Tc-MIBI-SPECT exhibited a high DOR. These nuclear medicine imaging techniques reflect cellular metabolism like amino acid transportation and transportation by P-glycoprotein; however, these were difficult to gain widespread use because of expensive specific apparatus and facilities. Conversely, the combination of DWI, MRS, and perfusion imaging exhibited the highest DOR among all imaging studies. Even with MRI, combined information with multiple parameters, including lesional metabolism and blood flow, enhanced the diagnostic accuracy, facilitating the differentiation between BRN and TP in conventional radiological imaging. In the subanalysis, Gd-enhanced MRI and ^18^F-FDG-PET revealed a low DOR and were useless to differentiate between BRN and TP in patients with glioma. In metastatic brain tumors, however, no difference was noted in the DORs among all radiological imaging methods. Hence, BRN could be diagnosed using any radiological imaging, such as Gd-enhanced MRI in metastatic brain tumors, and it is imperative to use specific imaging modality like combined imaging or new nuclear medicine for the diagnosis of BRN in gliomas.

In this review, many studies had a risk of bias. We included no randomized controlled trial, and only nine prospective cohort studies had a low risk of patient selection [18, 28, 29, 32, 36, 37, 39, 46, 47]. In addition, 26 (56.5%) studies were retrospective and had a bias to enroll a particular population of patients. In only two studies, a cutoff value for the best discrimination between BRN and TP was preset [22, 58]. Of note, retrospectively-set cutoff values could be overestimated and should be prospectively validated in future studies. Regarding the reference standard, histology was taken from all patients in only six studies (13%) [16, 17, 21, 30, 47, 48]. In studies using the clinical diagnosis as the reference standard, BRN was primarily if the clinical status and radiologically identified lesions were stable > 6 months. Hence, there was a possibility of confounding between the index test and the reference.

Regarding indirectness, various brain tumors were included. Reportedly, the development of radiation necrosis correlated with the total radiation dose, fraction size, treatment duration, and irradiated volume [61]; these factors of radiotherapy are different in applied radiotherapy between glioma and metastatic brain tumors. In addition, variable tumor cells and necrosis usually coexist in glioma after radiotherapy. Mixed lesions with tumor cells and necrosis render distinguishing between BRN and TP challenging even by histological examination. Thus, it is ideal to analyze the diagnostic accuracy of radiological imaging, dividing into glioma and metastatic brain tumors in the systematic review. Notably, diagnostic parameters were different among studies using the same imaging method. Moreover, when the same parameters were used for the same imaging method, the cutoff values were different among the studies, similar to those with L/N ratios for ^11^C-MET-PET. This, imprecision should be considered when assessing study results. In this review, strong evidence could not be obtained owing to the quantitative synthesis of studies with small sample size. We focused on PET with glucose and amino acid tracers as PET studies because several studies with these PET were published, which could be suitable for the meta-analysis. However, recent PET studies with new tracers, like ^18^F-DOPA, reported good results of differentiation between BRN and TP [62, 63]. In the near future, PET with new tracers would be investigated for the diagnostic accuracy in a meta-analysis after the adequate accumulation of studies. Recently, a PET/MRI study reported that FDG-PET/MRI could predict the local tumor control after stereotactic radiosurgery in patients with brain metastases [64]. Moreover, Jena et al. used PET/MRI for differentiating between BRN and TP in patients with glioma [65, 66]. Notably, PET/MRI can simultaneously evaluate lesions with several parameters including not only the tracer uptake but also ADC, chemical shifts, and CBV. Like the highest diagnostic accuracy of combination imaging with DWI, MRS, and/or perfusion MRI in this review, PET/MRI could exhibit high diagnostic accuracy in a future systematic review.

## Conclusions

In the systematic review for diagnosing BRN, 20 studies for conventional radiological imaging and 26 studies for nuclear medicine studies were identified. All studies had small sample size, and many carried a risk of bias and indirectness. This review reveals that it is difficult to draw a firm conclusion as to which is the best imaging study for the BRN diagnosis. In patients with glioma, Gd-enhanced MRI and ^18^F-FDG-PET were unlikely to diagnose BRN, although the diagnostic ability was almost the same among included imaging in metastatic brain tumors. Combined imaging methods that include metabolic and blood flow imaging methods demonstrated the highest DOR among all imaging studies. The development of multiparametric imaging techniques could enhance the diagnostic accuracy for differentiating between BRN and TP in the future.

## Additional files

Additional file 1: Searching key words for RQ1 (conventional radiological image) and RQ2 (nuclear medicine image). (DOCX 13 kb)

Additional file 2: Detail information about included studies in each radiological image. (DOCX 196 kb)
