# Supplementary material for: Radiological diagnosis of brain radiation necrosis after cranial irradiation for brain tumor: a systematic review
Source: Radiat Oncol. 2019 Feb 6;14:28. doi: 10.1186/s13014-019-1228-x (PMC6364413; doi:10.1186/s13014-019-1228-x)
Supplement: Supplementary file 1 — Searching key words for RQ1 (conventional radiological image) and RQ2 (nuclear medicine image). (DOCX 13 kb) [file 13014_2019_1228_MOESM1_ESM.docx]

Appendix. Searching key words for RQ1 (conventional radiological image) and RQ2 (nuclear medicine image)

Searching key words were follows; (“brain” OR “cerebral” OR “cerebrum” OR “central nervous system” OR “lobe”) AND (“radio necrosis” OR “radiation necrosis” OR “radiation injury” OR “brain lesion”) AND (“recurrence” OR “recurrent” OR “progression” AND “brain neoplasm” AND “tumor”) AND (“English [Lang]” OR “Japanese [Lang]”). For RQ1, additional key words were follows: (“MRI” OR “MR” OR “magnetic resonance” OR “CT” OR “computed tomography” AND “diagnosis, differential”) NOT (“PET” OR “positron emission tomography” OR “nuclear medicine” OR “radioisotope”). For RQ2, additional key words were follows: (“PET” OR “positron emission tomography” OR “positron emission tomograph”) AND “FDG” OR “fluorodeoxyglucose”) OR (“amino acid” OR “amino acids” OR “methionine”).
