# Supplementary material for: Radiological diagnosis of brain radiation necrosis after cranial irradiation for brain tumor: a systematic review
Source: Radiat Oncol. 2019 Feb 6;14:28. doi: 10.1186/s13014-019-1228-x (PMC6364413; doi:10.1186/s13014-019-1228-x)
Supplement: Supplementary file 2 — Detail information about included studies in each radiological image. (DOCX 196 kb) [file 13014_2019_1228_MOESM2_ESM.docx]

Detail information about included studies in each radiological image

*RQ1: Conventional Radiological Imaging*

*Gadolinium-enhanced MRI*

The diagnostic accuracy of Gd-enhanced MRI for BRN was evaluated in 4 studies [[16-19](#_ENREF_16)]. There were 2 studies that included patients with metastatic brain tumors and another 2 studies that included patients with glioma. In studies involving brain metastases, matching contrast-enhanced lesions with a lesion border visualized on T2-weighted imaging, or edema/lesion volume ratios were used as diagnostic parameters in each study [[16](#_ENREF_16), [17](#_ENREF_17)]. In studies of glioma patients, Gd-enhancement or multiple sequences for diagnosis was evaluated by radiologists [[18](#_ENREF_18), [19](#_ENREF_19)].

*Diffusion-Weighted Image*

DWI was used for diagnosis in 2 studies [[20](#_ENREF_20), [21](#_ENREF_21)]. In the study of patients with glioblastomas, the apparent diffusion coefficient (ADC) value was measured for diagnosis of BRN [[20](#_ENREF_20)]. In study including metastatic brain tumors, the layering pattern of ADC values was used for diagnosis of BRN.

*MR Spectroscopy*

There were 9 studies that used MRS to differentiate between BRN and TP [[20](#_ENREF_20), [22-29](#_ENREF_22)]. Of these, 6 studies featured patients with gliomas, and intracranial neoplasm, primary brain tumor, and metastatic brain tumor were included in one study each. In studies subjected to high-grade glioma or glioblastoma, values of Cho and N-acetylaspartate (NAA), Cho/Creatinine (Cr) and Cho/NAA, and Cho/ normal Cr were used as parameters for detection of BRN.[[20](#_ENREF_20), [27-29](#_ENREF_27)] In other studies, Cho/Cr, Lactate (Lac)/Cho, Cho/NAA were used for diagnosis [[22-24](#_ENREF_22), [26](#_ENREF_26)]. The cut-off values of these parameters and the associated measures of diagnostic accuracy are shown in Table 1.

*Perfusion CT and MRI*

Perfusion imaging for diagnosis of BRN was performed using CT in one study [[30](#_ENREF_30)] and MRI in 8 studies [[20](#_ENREF_20), [21](#_ENREF_21), [25](#_ENREF_25), [31-35](#_ENREF_31)]. In studies using perfusion MRI, 2 studies included metastatic brain tumors alone [[21](#_ENREF_21), [25](#_ENREF_25)] and 5 included glioma alone [[20](#_ENREF_20), [31-34](#_ENREF_31)]. The other study included various brain tumors [[35](#_ENREF_35)]. In studies of metastatic brain tumors, CBV was the best discriminator of BRN and TP [[21](#_ENREF_21), [25](#_ENREF_25)]. In glioma studies, CBV, relative peak height, and K^trans^ were used to differentiate BRN from TP [[20](#_ENREF_20), [31-34](#_ENREF_31)]. Although normalized relative CBV was most frequently used as a diagnostic parameter, the cut-off values were different among the 3 studies (Table 1) [[20](#_ENREF_20), [33](#_ENREF_33), [34](#_ENREF_34)].

*Combination of multiple imaging*

Three studies evaluated the diagnostic accuracy of combining multiple imaging techniques, including DWI and MRS, DWI and perfusion MRI, and DWI, MRS, and perfusion MRI used together to distinguish BRN from TP [[20](#_ENREF_20), [21](#_ENREF_21), [28](#_ENREF_28)]. All studies suggested that combining multiple imaging methods could help diagnose BRN more accurately than any single imaging study.

*RQ2: Nuclear Medicine Imaging*

*SPECT*

^201^Tl was used as a tracer in 6 studies [[19](#_ENREF_19), [36-40](#_ENREF_36)], ^99m^Tc-MIBI was used in 2 studies [[40](#_ENREF_40), [41](#_ENREF_41)], and ^99m^Tc-GHA was used in one study [[42](#_ENREF_42)]. In studies of ^201^Tl-SPECT, only 3 included glioma patients [[19](#_ENREF_19), [36](#_ENREF_36), [39](#_ENREF_39)], and another 3 studies featured glioma and metastatic brain tumors with or without other brain tumors [[37](#_ENREF_37), [38](#_ENREF_38), [40](#_ENREF_40)]. Tl index, visual assessment, and lesion/normal (L/N) ratio were used as diagnostic parameters in 2 studies [[38](#_ENREF_38), [39](#_ENREF_39)], 2 studies [[19](#_ENREF_19), [36](#_ENREF_36)], and one study [[40](#_ENREF_40)], respectively. The combination of visual assessment and L/N ratio was assessed by nuclear medicine specialists in one study [[37](#_ENREF_37)]. In 2 studies of ^99m^Tc-MIBI-SPECT, both studies used L/N ratio as a diagnostic parameter, but the optimal cut-off values differed between these 2 studies [[40](#_ENREF_40), [41](#_ENREF_41)].

*PET*

^18^F-FDG was used in 9 studies [[37](#_ENREF_37), [39](#_ENREF_39), [43-49](#_ENREF_43)] and ^11^C-MET was used in 8 studies [[48](#_ENREF_48), [50-56](#_ENREF_50)]. ^18^F-FET was used in 3 studies [[57-59](#_ENREF_57)]. ^18^F-BPA [[60](#_ENREF_60)] was used in only one study. In studies for ^18^F-FDG-PET, 4 studies included glioma alone [[39](#_ENREF_39), [46-48](#_ENREF_46)], and 2 studies included brain metastasis alone [[43](#_ENREF_43), [45](#_ENREF_45)]. Other 3 studies were subjected to various tumors [[37](#_ENREF_37), [44](#_ENREF_44), [49](#_ENREF_49)]. With regard to assessment, neuroradiologists visually assessed PET data in 7 studies [[37](#_ENREF_37), [39](#_ENREF_39), [43](#_ENREF_43), [44](#_ENREF_44), [46](#_ENREF_46), [47](#_ENREF_47), [49](#_ENREF_49)], and L/N ratio was used in 2 studies [[45](#_ENREF_45), [48](#_ENREF_48)]. In ^11^C-MET-PET studies, most of studies (5 studies) were subjected to glioma and brain metastases with or without other brain tumors [[50](#_ENREF_50), [52-55](#_ENREF_52)]. Two studies included glioma alone [[48](#_ENREF_48), [51](#_ENREF_51)] and one included brain metastasis alone [[56](#_ENREF_56)]. The L/N ratio was used to differentiate between BRN and TP in 4 studies [[48](#_ENREF_48), [50](#_ENREF_50), [53](#_ENREF_53), [54](#_ENREF_54)]. The other studies adopted visual assessment [[51](#_ENREF_51), [52](#_ENREF_52), [56](#_ENREF_56)] or uptake value [[55](#_ENREF_55)].
